# The Diagnosis and Therapy of Degenerative Knee Joint Disease: Expert Consensus from the Chinese Pain Medicine Panel

**DOI:** 10.1155/2018/2010129

**Published:** 2018-12-13

**Authors:** Dong Huang, Yan-Qing Liu, Li-Shuang Liang, Xue-Wu Lin, Tao Song, Zhi-Gang Zhuang, Suo-Liang Wang, Hong-Guang Bao, Lin Wang, Xian-Wei Zhang, Zhi-Gang Cheng, Bao-Lin Duan, Wei-Dong Qiu, Yuan-Chang Xiong, Jin-Feng Liu

**Affiliations:** ^1^The Third Xiangya Hospital of Central South University, Changsha, China; ^2^Beijing Tian Tan Hospital, Capital Medical University, Beijing, China; ^3^Qilu Hospital, Shandong University, Jinan, China; ^4^The First Affiliated Hospital of Bengbu Medical College, Bengbu, China; ^5^The First Affiliated Hospital of China Medical University, Shenyang, China; ^6^Algology Department, The Second Affiliated Hospital of Zhengzhou University, Zhengzhou, China; ^7^The First Affiliated Hospital of Xi'an JiaoTong University, Xi'an, China; ^8^Nanjing First Hospital, Nanjing Medical University, Nanjing, China; ^9^The First Affiliated Hospital of Guizhou Medical University, Guiyang, China; ^10^Tongji Hospital of Tongji Medical College, Huazhong University of Science and Technology, Wuhan, China; ^11^Xiangya Hospital, Central South University, Changsha, China; ^12^People's Hospital of Qinghai Province, Xining, China; ^13^The Second Affiliated Hospital of Zhejiang University School of Medicine, Hangzhou, China; ^14^Changhai Hospital, The Navy Military Medical University, Shanghai, China; ^15^The Second Affiliated Hospital of Harbin Medical University, Harbin, China

## Abstract

At present, there are many constantly updated guidelines and consensuses on the diagnosis and treatment of osteoarthritis both at home and abroad. The recommendations established using methods of evidence-based medicine has experienced strict research on controlling bias and promoting reproduction rate. As a result, the previous evidence was reevaluated, and a lot of changes were provoked in the diagnosis and treatment concept of osteoarthritis. However, several methods not recommended by foreign guidelines are still in use in the current clinical practice in China. On the one hand, Chinese experts have not reached extensive consensus on whether it is necessary to make changes according to foreign guidelines. On the other hand, almost all the current relevant guidelines are on osteoarthritis, but the lesions around knee joints which, as a whole, bear the largest weight in human body, cannot be ignored. For this purpose, Chinese Association for the Study of Pain (CASP) organized some leading experts to formulate this Chinese Pain Specialist Consensus on the diagnosis and treatment of degenerative knee osteoarthritis (DKOA) in combination with the guidelines in foreign countries and the expert experience of clinical practice in China. The consensus, which includes the definition, pathophysiology, epidemiology, clinical manifestation, diagnostic criteria, and treatments of DKOA, is intended to be used by first-line doctors, including pain physicians to manage patients with DKOA.

## 1. Overview

Degenerative knee osteoarthritis (DKOA) refers to a group of diseases caused by the degenerative changes and chronic cumulative damage of the knee joint, with knee articular cartilage denaturation and destruction, subchondral bone sclerosis or cystitis, hyperosteogeny of joints margin, synovium lesions, and/or the decompensated changes in surrounding tendons, ligaments, and other structures as the main pathological manifestations, knee joint pain, and movement restriction as the main clinical manifestations [1]. At present, there are many constantly updated guidelines and consensuses on the diagnosis and treatment of osteoarthritis both at home and abroad. The recommendations established using methods of evidence-based medicine have experienced strict research on controlling bias and promoting reproduction rate. As a result, the previous evidence was reevaluated, and a lot of changes were provoked in the diagnosis and treatment concept of osteoarthritis. For example, the Guideline on Evidence-Based Medicine for Knee Osteoarthritis (2nd edition) promulgated by American Academy of Orthopaedic Surgeons (AAOS) in 2013 pointed out that there is clear evidence that patients are not recommended for intra-articular injection of sodium hyaluronate, arthroscopic lavage, etc. However, such methods are still in use in the current clinical practice in China. On the one hand, Chinese experts have not reached extensive consensus on whether it is necessary to make changes according to foreign guidelines. On the other hand, almost all the current relevant guidelines are on osteoarthritis, but the lesions around knee joints which, as a whole, bear the largest weight in human body cannot be ignored. For this purpose, Chinese Association for the Study of Pain (CASP) organized some leading experts to formulate this Chinese Pain Specialist Consensus on the diagnosis and treatment of degenerative knee osteoarthritis (DKOA) in combination with the guidelines in foreign countries and the expert experience of clinical practice in China. The consensus is mainly to guide first-line doctors, including pain physicians, in the clinical diagnosis and treatment of DKOA.

## 2. Etiology and Pathogenesis

1. Risk factors such as age, obesity, gender, region, occupation, ethnicity, hormonal status, and genetic factors are associated with the morbidity of DKOA, among which age and obesity are of special importance. The specific pathogenesis of DKOA remains unclear. At present, it is generally agreed that the imbalance of anabolic and catabolic changes in chondrocytes, extracellullar matrix, and subchondral bone caused by both biomechanical and biological factors is one of the important pathogenic factors [1]. The main pathological changes are apoptosis of chondrocytes and abnormal extracellullar matrix. The type II collagen provides a meshwork that parallels to the articular surface, proteoglycans, and water are embedded within this framework, providing compressive resistance. As the joint is subjected to repeated and excessive loads, these meshwork changes and anticompression ability will be weakened, abnormal proliferation and apoptosis of the chondrocytes will be abnormally proliferated and apoptotic, and various cytokines, free radicals, and enzymes will be released. As a result, the pathological changes will be aggravated [2], causing cartilage damage and injury of the supporting structure [3]. Besides, metabolic factors such as hypertension, dyslipidemia, osteoporosis, and impaired glucose tolerance also affect the occurrence and development of the DKOA [4]. COL2A1 was the first mutant gene that has been found to be associated with the pathogenesis of DKOA [5], and after that, 10 more mutations were reported in succession [6].
2. Acute and chronic knee injuries substantially increase the risk of DKOA [7, 8], and it is considered that the severity of synovitis is associated with the progression of DKOA [9]. Anatomical abnormalities of the joint such as varus and valgus deformity may facilitate the development of DKOA and functional incapacitation [10], as a result of imbalance distribution of stress caused by anatomical changes [11]. Muscle weakness and ligamentous laxity which give rise to joint instability are also deemed to be the risk factors of DKOA [12], so exercise is beneficial to the treatment and remission of DKOA [13].
3. Periarthritis of knee is essentially an adaptive change after long-term load and damage, and this change can hardly compensate the original function. During the procedure, aseptic inflammation could be induced, that is why nonsteroidal anti-inflammatory drugs are effective. Within limits, adaptive change of the joint structure like tendon and ligament can increase the capability of the withstanding load. However, pathological changes will appear when the load is beyond compensation [14]. In the area with poor blood supply, ischemia and hypoxia can cause degeneration and necrosis, and at the same time, the expression of inflammatory mediators, enzymes, and vascular growth factors is upregulated, which lead to angiogenesis and tissue damage, inducing pain [15].

## 3. Epidemiology

The prevalence of symptomatic knee osteoarthritis (KOA) diagnosed by the clinician ranges from 4.2%–15.5%, increasing with ageing, and is associated with region, ethics, etc [16]. In Chinese population, the prevalence of symptomatic KOA was 8.1%, presenting a difference in sex (10.3% in women and 5.7% in men) and region (2 times higher in rural areas than in urban areas) [17]. The prevalence of KOA based on radiography is much higher, about 80% of the population above 65 years reveals radiographic evidence of KOA, and only 60% of those have symptoms [18, 19]. KOA, the leading cause of pain and disability worldwide, ranked 11th among 291 disease for disability globally [18, 19]. Almost 1/4 of KOA patients will suffer severe joint pain. Periarticular lesions and inflammation arose from synovium ligament, tendon, and muscle are major causes of knee osteoarthrosis, existing alone or coexisting with KOA. In population with the painful knee, the prevalence of gonarthromeningitis reached 67%, while bursal synovitis reached 0.2%–0.7% [20–22].

## 4. Classification and Clinical Characteristics of Degenerative Knee Osteoarthritis

According to the position of lesions, the classification and clinical characteristics of degenerative knee osteoarthritis are as follows:

1. Degenerative knee osteoarthritis [23]
2. Chondromalacia patellae [24]
3. Patellar tendonitis [25]
4. Subpatellar fat pad inflammation [26]
5. Medial collateral ligament inflammation [27]
6. Lateral collateral ligament inflammation [28]

## 5. Clinical Manifestation (Includes Symptom, Sign, Laboratory Examination, and X-Ray Examination)

### 5.1. Degenerative Knee Osteoarthritis

#### 5.1.1. Clinical Manifestation

DKOA is commonly seen in wrinkly and elderly people, and the main clinical symptom is arthralgia, which is manifested by recent recurrent knee joint pain [29]. Arthralgia is aggravated after moving or bearing weight, while relieves after rest. Local tenderness is an obvious symptom. Some patients have morning stiffness, but the symptom is no more than 30 minutes. With the disease progressing, the knee joint would be swollen, articular cartilage destruction, articular surface roughness, and bony crepitus (sense) may occur with joint activity. Joint deformity may appear as the patient's condition is further aggravated.

#### 5.1.2. Auxiliary Examination

Laboratory examinations show that the level of C-reactive protein (CRP) and/or erythrocyte sedimentation rate (ESR) are normal or slightly elevated. Articular cavity effusion (at least 2 times) is clear and sticky, and the leukocyte count is less than 2000 mL. The X-ray examination in standing or weight-bearing position shows that the joint space appears asymmetrical narrow, the subchondral bone appears sclerosis and cystic degeneration, and the joint marginal appears osteogenesis and forms osteophyte, and the joint may even appear deformity.

### 5.2. Chondromalacia Patellae

#### 5.2.1. Clinical Manifestation

Chondromalacia patellae is common in sports enthusiasts, most of whom has a history of trauma. Initial pain appears in the patella or knee. It is obvious at the beginning of activity, while alleviated quickly. But the pain will aggravate with prolonged exercise and then disappears after rest. When the condition is aggravated, the pain time is longer than that of the remission time. In severe cases, patients cannot be squatted and have trouble in step movement. Sometimes, patients appear lower extremity weakness suddenly and fall down. Physical examinations suggest that the prevalence of patellar edge tenderness in these patients is more than 90%. Grinding the patella in extending the position may result in patella friction and associated pain [30].

#### 5.2.2. Auxiliary Examination

In the early stage, there is no abnormality in the X-ray film. In the advanced stage, osteophyte would be formatted at the edge of the patella, the patella joint surface would be unsmooth, and the joint space would be narrow. Radionuclide bone imaging examination can reveal the localized radionuclides of the patella, which is essential for early diagnosis.

### 5.3. Patellar Tendinitis

#### 5.3.1. Clinical Manifestation

Pain and tenderness appear in the tibial tuberosity and the patellar ligament. When the illness state is slight, knee movement is normal, only with weakness and pain. With the illness state aggravating, patients feel difficult to go up and downstairs, and often keep knee joint in the flexed position because of straightening pain [31].

#### 5.3.2. Auxiliary Examination

X-ray shows sclerosis, roughness, and bone hyperplasia on patellar articular surface. MRI examination has a specific diagnostic value.

### 5.4. Subpatellar Fat Pad Inflammation

#### 5.4.1. Clinical Manifestation

Subpatellar fat pad inflammation most commonly occurred in mountain-climbing enthusiasts and long-term squatting position workers, who have a long history of knee joint strain. The disease develops slowly, and is manifested as pain in the knee joint and asthenia at the anterior inferior side of the knee. The tenderness appears in the lower margin of the patella and both sides of the patellar tendon. The knee joint pain aggravates with activity and alleviates after rest. With the patients condition worsened, the pain will be persistent and aggravated when squatting or going up and downstairs [32, 33].

#### 5.4.2. Auxiliary Examination

Knee joint X-ray examination can help exclude knee osteoarthritis, and MRI examination can help exclude knee joint meniscus and other injuries.

### 5.5. Knee Joint Medial Collateral Ligament Inflammation

#### 5.5.1. Clinical Manifestation

Knee joint medial collateral ligament inflammation is common in ball and snow athletes because of more jumping activities. Pain appears in the medial part of the knee when medial accessory ligament is damaged. When medial accessory ligament is partly ruptured, the pain sharpens and walking will be affected. When the ligament is completely ruptured, the pain will be significantly aggravated, and there will be muscle spasm, limitation of knee flexion or extension, and walking difficulties. Patients with medial accessory ligament inflammation have signs of tenderness at both starting and ending points of medial accessory ligament.

#### 5.5.2. Auxiliary Examination

The X-ray film shows normal findings. MRI could help to determine the degree of injury in the ligament and knee.

### 5.6. Knee Joint Lateral Collateral Ligament Inflammation

#### 5.6.1. Clinical Manifestation

The patients and their clinical manifestations are the similar to those people with knee medial accessory ligament injury. Knee joint lateral accessory ligament inflammation normally happens after injury. However, because of lateral accessory ligament disconnecting with joint cavity, it rarely causes joint effusion. The tenderness is obvious at both starting and ending points of lateral accessory ligament.

#### 5.6.2. Auxiliary Examination

The MRI examination could help to reflect the damage degree of ligament and effusion in the knee joint.

## 6. Diagnostic Criteria

### 6.1. Degenerative Knee Osteoarthritis

1. Recurrent episodes of genual arthralgia for one month [23, 34, 35]
2. X-ray examination shows asymmetric arthrostenosis, subchondral bone sclerosis and (or) cystic degeneration, and osteophytes of the joints
3. Synovial fluid (at least 2 times) is clear and viscous, and the leukocyte count is less than 2000 ml
4. Middle-aged patients (over 40 years of age)
5. The time of morning stiffness is less than 30 min
6. Bony crepitus occurs with joint activity
7. The level of ESR or CRP is normal or slightly high [36]

Degenerative knee osteoarthritis can be diagnosed by fulfilling following conditions: (1) + (2) or (1) + (3) + (5) + (6) or (1) + (4) + (5) + (6). (7) is for reference.

Imaging classification (Kellgren–Lawrence level).

  Grade 0: normal
  Grade I: doubtful narrowing of the joint space with possible osteophyte formation
  Grade II: possible narrowing of the joint space with definite osteophyte formation
  Grade III: definite narrowing of joint space, moderate osteophyte formation, and some sclerosis
  Grade IV: large osteophyte formation, severe narrowing of the joint space with marked sclerosis, and definite deformity of bone ends

**Figure 1:**
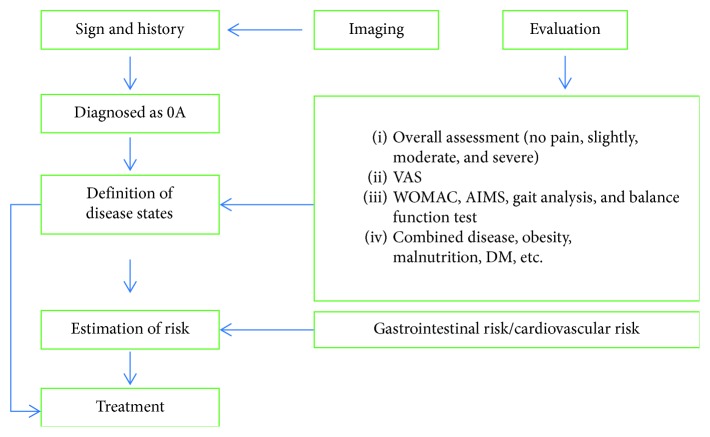


Diagnostic and evaluation flow chart is shown in Figure 1 [23].

### 6.2. Chondromalacia Patellae

1. Pain at the anterior knee or posterior patella, obviously in the squat position [37, 38]
2. Asthenia of the knee joint was obvious when walking up or downstairs
3. Patellar tenderness
4. Patella pressure test (+)
5. Single leg squat test (+)
6. X-ray examination shows hyperostosis. Axis examination shows patellar tilt or subluxation, narrowing of lateral joint space

Chondromalacia patellae can be diagnosed by following conditions: meeting (1) + (2) + (3) + (4) + (5) or (2) + (3) + (4) + (5) + (6).

### 6.3. Patellar Tendonitis

1. Pain at jumping or squatting and no pain at walking [39]
2. Pain at the beginning of movement, reduced or disappeared after activities, and feeling fatigued pain after exercise
3. Patellar tendon tenderness
4. Local swelling
5. B-mode ultrasonography shows the signs of patellar tendon inflammation [40]

Patellar tendonitis can be diagnosed by fulfilling following conditions: (1) + (2) + (3) + (4) or (1) + (2) + (3) + (5).

### 6.4. Subpatellar Fat Pad Inflammation

1. Genual pain, aggravated when straightening the knee. Have difficulty to go downstairs, with the feeling of asthenia [41]
2. Xiyan (EX-LE4,5) swelling
3. Patellar tendon and patellar tip surface tenderness
4. Hyperextension test of the knee joint (+)
5. Patellar tendon relaxation test (+)
6. The X-ray film shows normal finding or a moderate degree of hyperostosis

Subpatellar fat pad inflammation can be diagnosed by fulfilling the following conditions: (1) + (2) + (3) + (4) or (1) + (2) + (3) + (5). (6) is for reference.

### 6.5. Medial Collateral Ligament Inflammation

1. Medial of knee joint pain, obviously at walking or squatting [42]
2. Medial collateral ligament tenderness (+)
3. Medial collateral ligament local swelling
4. Lateral compression test (+)
5. X-ray examination will show specific finding only when calcification of ligament and ossification are formed

Medial collateral ligament inflammation can be diagnosed by fulfilling the following conditions: (1) + (2) + (3) + (5) or (1) + (2) + (4) + (5) conditions can diagnose medial collateral ligament inflammation.

### 6.6. Lateral Collateral Ligament Inflammation

1. Lateral of knee joint pain [42]
2. Lateral collateral ligament tenderness (+)
3. Lateral compression test (+)
4. X-ray examination shows calcification of ligament and ossification

Medial collateral ligament inflammation can be diagnosed by fulfilling the following conditions: (1) + (2) + (3) or (1) + (2) + (3) + (4).

It is recommended to use MRI for auxiliary examination, and MRI combined with clinical symptoms and signs can help definitive diagnosis and staging of disease.

## 7. Differential Diagnosis

### 7.1. Knee Rheumatoid Arthritis

Knee rheumatoid arthritis is a synovial inflammation disease of the knee caused by various causes. Chronic inflammation of the synovial membrane leads to hyperplasia of the knee joint, erosion of articular cartilage and ligaments, etc., eventually resulting in deformity and loss of function. Knee rheumatoid arthritis usually affects middle-aged women, and its typical symptoms are symmetrical polyarthritis, morning stiffness, and rheumatoid nodules around the knee joints in severe patients. A laboratory test reveals continuing positive in the rheumatoid factor. Magnetic resonance imaging is more sensitive in the early diagnosis of rheumatoid arthritis due to its high tissue resolution [43].

### 7.2. Rheumatoid Arthritis

Rheumatoid arthritis is closely related to human hemolytic streptococcal infection. The most common clinical manifestations of rheumatoid arthritis are knee pain, myalgia, muscle weakness, increased muscle enzymes, myogenic damage, etc. Irregular fever without chills appears before rheumatism and reveals insensitive to antibiotic therapy, and there are some abnormities in the blood test of autoantibody, such as anti-ENA antibodies, anti-ds-DNA antibodies, anti-platelet antibodies, anti-nuclear antibodies, and anti-cardiolipin antibodies. In the acute phase of the disease, blood sedimentation rate can reach 90 mm/hr or more; the level of C-reactive protein exceeds 30 mg/L (30 *μ*g/mL) and then gradually returns to normal after 1-2 months. The proportion of males and females is nearly equal, and the initial episode affected is more common in 9–17-years-old. The musculoskeletal ultrasound for rheumatic osteoarthropathy in the children can provide an accurate theoretical basis for the diagnosis of the disease [44].

### 7.3. Posttraumatic Knee Arthritis

The knee joint is the site most susceptible to injury. Posttraumatic knee arthritis is a disease with main clinical manifestations of joint pain and dysfunction, which may occur in any age group, with a history of knee trauma, pain, and even swelling. Its main pathological changes cover traumatic degeneration of articular cartilage and secondary cartilage hyperplasia, ossification. There are no specific laboratory tests for traumatic arthritis. Some X-ray findings of the knee joint include fractures or malunion lines, space narrowing, subchondral articular sclerosis, and bone spur formation, and at late phase, unsmooth joint surface, bone deformation, and intracapsular corpus liberum [45]. Pain may be induced by activities, and patients may feel a sensation of rough friction when moving the knee joint. However, X-ray imaging shows joint deformation and locked joint.

### 7.4. Tuberculosis of the Knee Joint

The majority of tuberculosis of the knee joint are secondary to pulmonary tuberculosis, and however, some of them are primary [46]. Generally tuberculosis of the knee joint is of bone type, and the synovium type may exist when the synovial membrane is attacked by hematogenous infections. X-ray imaging of bone type of joint tuberculosis is characterized by asymmetry of the joint space narrowing or destruction of the joint bone and periarticular soft tissue swelling. Synovium type of joint tuberculosis progresses slowly, with bone destruction limited to the edge of the articular surface, which gradually affects the weight-bearing part of knee. The early X-ray examination shows swelling of the joint capsule and articular soft tissue, increased density, normal or widened joint space, and osteoporosis. All these changes are attributed to the formation of granulation tissue and joint effusions arose from swelling and thickening of the synovial membrane. High-frequency ultrasound can show the degrees of synovial thickness, effusion, cartilage, and bone damage in the tuberculosis of the knee joint, having an important value for the diagnosis [47].

### 7.5. Infectious Arthritis of the Knee

The clinical manifestations of warmth, pain, swelling, and redness and the microbiological examination of the infection site are helpful for diagnosis. In most cases, laboratory tests reveal ESR accelerating, white blood cell count, and C-reactive protein level increasing. WBC count in the synovial fluid sample of the acute infected swollen joint was >20000 *μ*l, and the neutrophils rate was >95%. The viscosity and sugar concentration of synovial fluid decreased. Gram-negative and Gram-positive bacteria in 50% to 75% of the infected joint can be identified by Gram staining. Synovial fluids also require anaerobic and aerobic cultivations. Anaerobic infection can be confirmed from the odor in synovial fluid, gas shadows in joints, or periarticular soft tissue under X-ray imaging. The latest literature proved that procalcitonin has a good efficacy in the diagnosis [48].

## 8. Prevention and Health Education

Patient education of knee osteoarthritis has been carried out to make patients fully aware of the natural progression of the disease, the purpose of the treatment, the usage and side effects of the medication as well as to minimize psychological burdens. By strengthening self-management, we can significantly improve the quality of life for these patients.

### 8.1. Self-Management and Countermeasures

To facilitate chronic care management for degenerative knee osteoarthropathy [49, 50], it is essential to emphasize the coparticipation of doctors and patients. This supports patients in developing an efficient self-management scheme. It is furthermore important to educate patients about the disease's etiology, prevention, and treatment. Through the general clinic, standardized telephone follow-up, consulting [51], health examination etc., a high risk population for knee osteoarthropathy will be selected for dynamically tracking the current situation and trend of health change in order to achieve the best preventative care measures.

### 8.2. Daily Care

It is important to pay attention to the protection of hurt joints at an early stage in order to avoid further damage. Lifestyle changes such as avoiding stair-climbing and crawling for long periods of time can occur. It is advisable to choose a combination of exercises to reduce the frequencies and lasting time of pain. Furthermore, since changes of weather may increase pain, damp as well as cold should be avoided.

### 8.3. Keeping a Healthy Weight

Patients with BMI > 25 should adopt a low caloric diet as well as get into the habit of performing aerobic exercise [52]. Excessive knee bearing should be avoided in order to reduce the risk of joint degeneration.

### 8.4. Walking with an Electronic Monitor

Slow walking under guidance of an electronic monitor is advised to activate cartilage metabolism and prevent muscular atrophy [53].

### 8.5. Psychological and Social Support

Health care staff may give psychological and social support to release the psychological burden of elderly people and increase the compliance of disease treatment.

### 8.6. Doctor-Patient Communication

Full communication between health care staff and patients on etiology and prognosis of their disease, and comprehensive information about the purpose, curative effect, and rehabilitation measures before and after the treatment with drugs or surgical operations, can greatly improve the compliance of the patients and the prognosis of the disease treatment.

## 9. Therapies

### 9.1. Nonpharmaceutical Therapies

Individualized rehabilitation evaluation, including the items of knee joint activity, lower limb muscle and muscle strength, degree of pain, and local soft tissue condition, should be implemented for patients with degenerative knee joint disease, through which comprehensive rehabilitation treatment plan can be formulated.

#### 9.1.1. Physiotherapeutics

Physiotherapy for degenerative knee joint disease may reduce pain and joint stiffness, eliminate inflammation, enhance the stability of muscles and joints, and improve joint function and the ability of daily activities [54, 55].

*(1) Ultrashort Wave Therapy*. The treatment of degenerative knee joint disease with ultrashort wave therapy can help reduce knee joint pain but has little effect on knee function improvement. Microheat and tepid are commonly chosen in ultrashort wave treatment.

*(2) Medium- and Low-Frequency Electric Therapies*. Medium- and low-frequency electric therapies are used to treat chronic inflammation, adhesion, muscular atrophy, and joint stiffness. Transcutaneous electrical neural stimulation is an effective way to control pain.

*(3) Ultrasound Therapy*. Ultrasound therapy can significantly relieve knee pain and partially improve the knee joint function in DKOA patients. Ultrasound therapy is used for the treatment of joint swelling and adhesion.

*(4) Shockwave Therapy*. Extracorporeal shockwave therapy for degenerative knee joint disease can help relieve knee pain and improve the knee function. The efficacy of moderate energy is better than that of low energy.

*(5) Infrared Treatment and Magnetic Therapy*. Low energy red light and infrared-ray therapy can help relieve pain and improve knee functions in patients with DKOA. Magnetic therapy is used to treat chronic inflammation such as arthritis and soft tissue inflammation around the joints [56].

*(6) Millimeter Wave Therapy*. Millimeter wave therapy reduces knee pain score and improves joint activity.

#### 9.1.2. Rehabilitation Therapy

Rehabilitation therapy relieves pain and improves joint activity [57]. Patient education, exercise, and body mass reduction are the economic first-line treatment programs [58, 59].

*(1) Exercise Therapy*. Exercise therapy mainly focuses on the function of muscle and joint activity [60], which is an important measure to prevent joint degeneration [61, 62]. Exercise therapy includes the following: (1) muscle strength training: strength training for quadriceps, hamstring, hip abductor group, and hip adductor group; (2) joint activity training: from nonweight-bearing to weight-bearing joint activity training; (3) body fitness training (aerobic exercise); (4) walking under the monitoring system.

*(2) Spa*. Arthritis of different ages can be treated with warm water for rehabilitation, which has obvious advantages and great safety [63, 64].

*(3) Application of Walking Aids*. Walking aid instrument can be appropriately used to alleviate the knee load for patients suffering from degenerative bone joint disease of the lower extremities.

### 9.2. Pharmacological Treatment

The main purpose of pharmacological therapy is to relieve pain, reduce inflammatory response, and improve joint function and the quality of life in the patients with degenerative knee osteoarthritis. The commonly used drugs are divided as follows:

#### 9.2.1. Acetaminophen

As a kind of acetanilide antipyretic analgesics, acetaminophen only relieves a mild-moderate pain. This product has no obvious anti-inflammatory effect, and the patients with mild pain can be used as the preferred drug for short-term use. Major adverse reactions include gastrointestinal symptoms and hepatotoxicity. Patients with severe liver and kidney dysfunction and patients allergic to acetaminophen are prohibited to use it [65–67].

#### 9.2.2. Nonsteroidal Anti-Inflammatory Drugs (NSAIDs)

NSAIDs can be recommended as the first-line drugs in the treatment of DKOA. Of note, NSAIDs should be used with caution in patients who are the elderly or has a history of asthma induced by aspirin, hepatorenal impairment, and coagulative dysfunction. NSAIDs are contraindicated and should be avoided in patients with upper gastrointestinal hemorrhage or perforation. Therefore, the patients should be fully evaluated before using these medicines and following the principle with minimum effective dosage, short course of treatment, and individualization as well.

*(1) Oral NSAIDs*. The selective NSAIDs could be more targeted to inhibit COX-2 enzyme, and the risk of adverse reaction of the digestive system was lower than that of nonselective NSAIDs. The representative drug is celecoxib.

*(2) Topical NSAIDs*. Topical NSAIDs could directly be used in the pain site and can quickly exert analgesic effect by penetrating into the local tissue, which can get a high local analgesic dose. Also, compared with Oral NSAIDs, topical NSAIDs have slighter systematic adverse reaction risks. The common adverse reactions for topical NSAIDs are erythema, itching, dryness, etc. These symptoms can be self-relieved [65–70].

*(3) Intravenous NSAIDs*. Intravenous NSAIDs could be used in patients who are unable to take oral medication or need intravenous fluids. The representative drug is ibuprofen injection.

#### 9.2.3. Opioids

It will be applied for the patients with no response to other analgesics or for the patients with moderate to severe acute and chronic pain. The use of opioids should follow the principle of individuality. Common adverse reactions include dry mouth, nausea, vomiting, dizziness, lethargy, and constipation. The overdose of opioids can cause respiratory depression. Tramadol should not be used in conjunction with 5-hydroxytryptamine drugs in order to avoid the occurrence of 5-hydroxytryptamine syndrome [68, 69].

#### 9.2.4. Glucosamine and Chondroitin Sulfate

Glucosamine is often used in dietary adjuvant therapy for osteoarthritis and can be combined with nonsteroidal anti-inflammatory drugs [71, 72]. However, the efficacy remains controversial. Some patients may experience gastrointestinal discomfort, lethargy, headache, and skin response. Patients allergic to shellfish are prohibited to take glucosamine.

Chondroitin sulfate can be used as an adjuvant for chronic pain. However, chondroitin sulfate can increase the risk of hemorrhage. Therefore, the patients taking anticoagulant should use such drugs with caution [73].

#### 9.2.5. Glucocorticoid

Intra-articular or pain points glucocorticoids injection may be used to manage acute pain and localized inflammation associated with DKOA, or the pain which is unresponsive to oral or external analgesics. Glucocorticoid can inhibit the protein polysaccharide synthesis in articular cartilage, and long-term use in the articular cavity can aggravate the damage of articular cartilage and aggravate the symptoms. Thus, using articular cavity injection of glucocorticoid arbitrarily is not recommended. As a general rule, the number of injection is limited to three for a single joint per year. The patients with severe mental history, active gastrointestinal ulcer, recent gastrointestinal anastomosis, severe osteoporosis, diabetes, hypertension, and infection are prohibited to use glucocorticoid [65, 74, 75].

#### 9.2.6. Sodium Hyaluronate

Its efficacy is still in controversial. However, it is recommended for early and medium to use for the patients with DKOA.

#### 9.2.7. Vitamins

Due to the antioxidation effect, Vitamins A, C, and E are beneficial for the treatment of DKOA. By the way, vitamin D exerts the therapeutic effect by bone mineralization and cell differentiation [76].

### 9.3. Traditional Medicine

Traditional medicine includes traditional Chinese medicine, Tibetan medicine, needle knives, silver needles, internal thermal needles, acupuncture, and massage.

Chinese medicine doctors believe that osteoarthritis is a “bone impediment.” The main reason is the deficiency of liver, spleen, and kidney, causing the local joints and tendons affected by wind, cold, and wet. Clinically, they often fall into the category of marrow depletion due to kidney deficiency, yang deficiency due to yin excess, and blood stasis [77]. Traditional medicine therapies can be listed as follows:

1. Internal application of traditional Chinese medicine. Commonly used proprietary Chinese medicines include Huoluo Pills, Duhuojisheng Pills, Zhuangyao Jianshen pills, and Zhuifeng Tougu Pills [78]. Clinically, patients taking Chinese patent medicines orally are advised to regularly test liver and kidney functions to ensure the safety of medication.
2. External application of traditional Chinese medicine. External application of traditional Chinese medicine, with rare adverse reactions, can significantly relieve mild-to-moderate joint pain. Commonly used methods include Chinese herbal fumigation and washing therapy, Chinese herbal iontophoresis, and Chinese herbal plaster. As herbal analgesics, several Tibetan medicines like Qizheng pain-relieving plaster and Qingpeng ointment exert anti-inflammatory and analgesic effects to help alleviate osteoarthritis pain and discomfort effectively [79]. The main mechanism is that those herbal analgesics can inhibit the abnormal immune activity of the pain pathway [80], inhibit the proinflammatory effects of various cytokines, and delay the destruction of the cartilage matrix.
3. Cupping therapy. Cupping can alleviate joint pain and stiffness and improve the quality of daily life and general condition [81, 82]. Especially when induration and cords in joints can be palpable, or ache, tenderness, numbness, and other pathological features are existed, cupping therapy combined with stabbing is effective.
4. Acupuncture therapy. Acupuncture, a nonpharmacological treatment, with few side effects, great safety, and effectiveness, is recommended as a basic therapy for osteoarthritis [83]. Ashi points and local acupoints are often chosen to implement warm acupuncture moxibustion, direct moxibustion, or suspended moxibustion. For severe joint pain, especially in cold cases, warm needle or fire needle therapy is recommended.
5. Needle knives therapy. Needle knife is a kind of therapeutic tool integrated traditional Chinese medicine and western medicine. The main mechanism of needle knife therapy is to relieve the local myofascial attachment points. By relieving the dynamic imbalance and restoring the mechanical balance of joint, the therapeutic effect can be achieved. For the muscle fascia adhesion, joint function limitation, and obvious deformity, acupuncture treatment is recommended. The choice of the site of administration is at the junction of pain points or tender points around the joints, or at sites of hyperosteogeny of the joints. The treatment was performed once a week, up to 3 times [84, 85].
6. Silver needles therapy. The silver needle originates from the ancient “Nine-Needles”, and it has the function of “treating other areas” and is “good for joints.” On account of the unique acupuncture and heat transfer diffusion effect of deep tissue, it produces anti-inflammatory and analgesic effects, improves blood supply, and relaxes muscle contracture. It has a significant long-term effect on chronic pain. Myofascial start and end points, fascial space, and local acupoints around the joints can be selected as the treatment sites of osteoarthritis. The treatment was performed once per week, and two or three times constituted one course of treatment [86, 87].
7. Internal thermal needles therapy. The internal thermal needle is made on the basis of traditional acupuncture and silver needles, and its mechanism of action is similar to silver needles. Due to the stainless steel material, the thermal conductivity diffusion effect in the deep soft tissue is not as good as that of the silver needle. The efficacy and mechanism remain to be observed.
8. Massage therapy. Massage has the effect of activating the circulation, relaxing the muscles and promoting blood circulation, reducing swelling and pain, smoothing the joints, and improving the muscle strength and joint function. It can be combined with other methods.

### 9.4. Minimally Invasive Therapy

Minimally invasive treatment guided by imaging is an effective method for the treatment of knee osteoarthropathy.

#### 9.4.1. Injection Therapy

Injection is a common method for the treatment of knee osteoarthropathy. For moderate and severe pain, trigger point injection, nerve block, and intra-articular injection can be used. Trigger point injection (local anesthetics + corticoid) can quickly relieve pain in patients with degenerative knee osteoarthritis accompanying ligament and tendon injury or inflammation but is not recommended to be used frequently or for a long time and as a first-line treatment method [88–92]. The injection of glucocorticoid and sodium hyaluronate into a point injection has been controversial. Most guidelines recommend the former [93–96]. Glucocorticoid is recommended when it is joint effusion or resting pain [97]. PRP [98–100] and ACS [101–103] have been used more in recent years and can be used in articular injection [97, 104]. Especially, it is safe and effective for PRP to be injected into the articular cavity for the treatment of osteoarthritis [105]. The more mature methods of nerve block for knee osteoarthritis are mainly femoral nerve [106], adductor canal, and saphenous nerve block [107–110]. Contraindications should be strictly controlled in injection therapy, especially in intra-articular injection [111]. Patients with local skin diseases at the injection site, diabetes mellitus, high blood pressure, and mental illness should be implemented with caution. The local or total body infection and low immune function are contraindications. If trigger point injection has been implemented for 2∼3 times and the pain was not relieved, alternative treatment strategies should be considered.

#### 9.4.2. Radiofrequency Therapy

Indications are as follows: (1) knee OA with moderate to severe pain and no response to at least 3–6 months of conservative treatment; (2) persistent pain after total knee arthroplasty (TKA) and conservative treatment [112, 113].

Contraindications are as follows: acute knee pain, bleeding tendency, localized infection, systemic infections, neurologic or psychiatric disorders, pregnancy, and pacemakers.

Procedures and adverse reactions:

1. RFS (standard radiofrequency): RFS can be performed under X-ray or ultrasonic imaging guidance, with a cannula advanced into the joint towards the area connecting the shaft to the epicondyle. The area is stimulated to identify the nerve position and to ensure that no motor nerves are activated, as evidenced by absence of fasciculations. The RF electrode is then advanced through the cannula to the target area. The electrode tip heats up targeted local tissue within a few millimeters to a temperature typically greater than 65∼90°C for 120∼130 seconds. Adverse effect is as follows: prolonged (2–6 weeks) hypoesthesia in the medial aspect of the knee was observed.
2. RFP (pulsed radiofrequency): The pulse generator produces pulses with an amplitude of 45 V lasting 20 milliseconds every 500 milliseconds (twice per second). The tissue temperature reaches a maximum of 42°C. Targets are as follows: (a) intra-articular: the entry point for the RF cannula was the anterolateral aspect of the knee midway between the femoral and tibial surfaces. RFP lasts for 10–15 min. (b) Major nerves or their main branches: femoral and sciatic nerves or their main branches (saphenous, tibial, and common peroneal nerves) were targeted. RFP lasts for 4–8 min. No adverse reactions were reported.

#### 9.4.3. Intra-Articular Ozone Injection Therapy

Indications are as follows: knee OA pain, Kellgren–Lawrence grade 2-3 [114–117].

Contraindications are as follows: ozone allergy, hyperthyroidism, G-6-PD deficiency, recent myocardial infarction, bleeding tendency, and pregnancy. Be cautious when hepatic and renal insufficiency were present.

Procedure and adverse reactions are as follows: intra-articular applications with an ozone concentration of 20–30 mg/L and volumes between 5 and 15 mL once a week for 3-4 weeks. Adverse reactions like transient pain when injecting was reported.

#### 9.4.4. Cryotherapy

Cryotherapy following total knee replacement (TKR): application of cold temperatures to the skin around the knee following surgery by means of ice packs, cooling pads, or other commercial devices within 48 hours after surgery, either continuously or replacing ice packs every 1.5–4 hrs [118]. Blood loss and pain could be improved mildly. Adverse reactions include discomfort, local skin reactions, superficial infection, cold injury, and venous thrombosis.

Cryoneurolysis of infrapatellar branch of the saphenous nerve (IPBSN): (1) indication: knee OA with moderate to severe pain, Kellgren–Lawrence grade 2-3, ≥50% reduction in VAS pain score when performing the activity that elicited the worst pain following a diagnostic lidocaine block of the IPBSN; (2) contraindication: gross deformity of the knee and body mass index (BMI) ≥35 kg/m^2^; (3) procedure and adverse effects: inject nitrous oxide with iovera device to form a highly localized cold zone via the Joule–Thompson effect. Low temperatures (−20°C to −100°C) to peripheral nerves cause Wallerian degeneration. Adverse effects include altered sensation, bruising, crusting, hyperpigmentation, itching, local pain, numbness, redness, swelling, tenderness on palpation, and tingling.

### 9.5. Surgical Management

Patients with severe lesions and obvious dysfunction of the knee who failed the treatments above may be considered to have surgical management, which includes the following: (1) knee arthroscopy [119, 120]: arthroscopic intra-articular lavage is performed to remove fibrin, cartilage residues, and other impurities if other minimally invasive treatments were invalid; arthroscopy with partial meniscectomy, joint debridement, or a combination of both techniques may be effective in patients who had mild-to-moderate osteoarthritis of the knee with a varus or valgus angle <5°; and however, the conventional application of arthroscopy was not supported by most of the literature evidences. There are adverse reactions such as deep vein thrombosis, pulmonary embolism, infection, and even death. (2) Osteotomy [121]: it can improve the balance of the joint force line and effectively relieve the knee pain in patients. (3) Arthroplasty [122–124]: progressive degenerative knee osteoarthritis in patients over 60 years with poor conservative management may be treated with joint replacement, which can significantly alleviate pain and improve the physical function of the knee. (4) Arthrodesis [125].

## References

[1] Glyn-Jones S., Palmer A. J. R., Agricola R. (2015). Osteoarthritis. *The Lancet*.

[2] Henrotin Y., Addison S., Kraus V., Deberg M. (2007). Type II collagen markers in osteoarthritis: what do they indicate?. *Current Opinion in Rheumatology*.

[3] Arden N., Nevitt M. (2006). Osteoarthritis: epidemiology. *Best Practice & Research Clinical Rheumatology*.

[4] Yoshimura N., Muraki S., Oka H. (2012). Accumulation of metabolic risk factors such as overweight, hypertension, dyslipidaemia, and impaired glucose tolerance raises the risk of occurrence and progression of knee osteoarthritis: a 3-year follow-up of the ROAD study. *Osteoarthritis and Cartilage*.

[5] Alakokko L., Baldwin C. T., Moskowitz R. W., Prockop D. J. Single base mutation in the type II procollagen gene (COL2A1) as a cause of primary osteoarthritis associated with a mild chondrodysplasia.

[6] Sandell L. J. (2012). Etiology of osteoarthritis: genetics and synovial joint development. *Nature Reviews Rheumatology*.

[7] Wilder F. V., Hall B. J., Barrett J. P., Lemrow N. B. (2002). History of acute knee injury and osteoarthritis of the knee: a prospective epidemiological assessment. *Osteoarthritis and Cartilage*.

[8] Toivanen A. T., Heliövaara M., Impivaara O. (2009). Obesity, physically demanding work and traumatic knee injury are major risk factors for knee osteoarthritis—a population-based study with a follow-up of 22 years. *Rheumatology*.

[9] Krasnokutsky S., Attur M., Palmer G., Samuels J., Abramson S. B. (2008). Current concepts in the pathogenesis of osteoarthritis. *Osteoarthritis and Cartilage*.

[10] Sharma L. (2001). The role of knee alignment in disease progression and functional decline in knee osteoarthritis. *JAMA*.

[11] Harris W. H. (1987). Etiology of osteoarthritis of the hip. *Clinical Orthopaedics & Related Research*.

[12] Cyrus Cooper M., Javaid K., Arden N. (2014). *Epidemiology of Osteoarthritis*.

[13] Bennell K. L., Wrigley T. V., Hunt M. A., Lim B.-W., Hinman R. S. (2013). Update on the role of muscle in the Genesis and management of knee osteoarthritis. *Rheumatic Disease Clinics of North America*.

[14] Magnusson S. P., Langberg H., Kjaer M. (2010). The pathogenesis of tendinopathy: balancing the response to loading. *Nature Reviews Rheumatology*.

[15] Michele A., Karin G. S., Carl S. (2009). Pathogenesis of tendinopathies: inflammation or degeneration?. *Arthritis Research & Therapy*.

[16] Cisternas M. G., Murphy L., Sacks J. J., Solomon D. H., Pasta D. J., Helmick C. G. (2016). Alternative methods for defining osteoarthritis and the impact on estimating prevalence in a US population-based survey. *Arthritis Care & Research*.

[17] Tang X., Wang S., Zhan S. (2016). The prevalence of symptomatic knee osteoarthritis in China: results from the China health and retirement longitudinal study. *Arthritis & Rheumatology*.

[18] GBD 2015 Disease and Injury Incidence and Prevalence Collaborators (2016). Global, regional, and national incidence, prevalence, and years lived with disability for 310 diseases and injuries, 1990-2015: a systematic analysis for the Global Burden of Disease Study 2015. *The Lancet*.

[19] Barbour K. E., Boring M., Helmick C. G., Murphy L. B., Jin Q. (2016). Prevalence of Severe Joint Pain Among Adults with Doctor-Diagnosed Arthritis—United States, 2002–2014. *Morbidity and Mortality Weekly Report*.

[20] Kim I. J., Kim D. H., Song Y. W (2016). The prevalence of periarticular lesions detected on magnetic resonance imaging in middle-aged and elderly persons: a cross-sectional study. *BMC Musculoskeletal Disorders*.

[21] Wang X., Jin X., Han W (2016). Cross-sectional and longitudinal associations between knee joint effusion synovitis and knee pain in older adults. *Journal of Rheumatology*.

[22] Le Manac’h A. P., Ha C., Descatha A., Imbernon E., Roquelaure Y. (2012). Prevalence of knee bursitis in the workforce. *Occupational Medicine*.

[23] Chinese Orthopaedic Association (2007). The guideline of diagnosis and treatment of osteoarthritis. *Chinese Journal of Joint Surgery (Electronic Version)*.

[24] WU Yu-J., Jian-Guo F. A. N. G. (2006). Progress of research on chondromalacia patellae. *Orthopedic Journal of China*.

[25] Rath E., Schwarzkopf R., Richmond J. C. (2010). Clinical signs and anatomical correlation of patellar tendinitis. *Indian Journal of Orthopaedics*.

[26] Figueroa D., Figueroa F., Calvo R. (2016). Patellar tendinopathy: diagnosis and treatment. *Journal of the American Academy of Orthopaedic Surgeons*.

[27] Waldman S. D. (2006). *Pain Managemet*.

[28] Yan-Qing L. I. U., Jin-Feng L. I. U., Li-Juan L. (2006). *The Algology Manual of Pain Medicine, Skeletal Muscle and Joint Volume*.

[29] Gao-Rong N. I., Xiao-Mei Z. H. A. N. G. (2016). A review: the safety of treatment of knee osteoarthritis by Ozone. *Chinese Journal of Pain Medicine*.

[30] Tian-Zun T. A. O. (2008). *New Practical Orthopaedics*.

[31] Maffulli N., Oliva F. (2017). The royal london hospital test for the clinical diagnosis of patellar tendinopathy. *Muscles, Ligaments and Tendons Journal*.

[32] Bode G., Hammer T., Karvouniaris N., Feucht M. J. (2017). Patellar tendinopathy in young elite soccer- clinical and sonographical analysis of a German elite soccer academy. *BMC Musculoskeletal Disorders*.

[33] Gang Y. E., Guo-Dong H. A. N., Yan-Li S. H. I. (2017). A clinical study of thermal therapy by silver needles for knee infra-patellar fat pad lesion. *Chinese Journal of Pain Medicine*.

[34] Chinese Rheumatology Association (2010). The guideline of diagnosis and treatment of osteoarthritis. *Chinese Journal of Rheumatology*.

[35] McAlindon T. E., Bannuru R. R., Sullivan M. C. (2014). OARSI guideline for Noe-surgical management of knee OA. *Osteoarthritis and Cartilage*.

[36] Kellgren J. H., Lawrence J. S. (1957). Radiological assessment of osteoarthrosis. *Annals of the Rheumatic Diseases*.

[37] Mian-Yu Q. U., Chang-Long Y. (2003). *Sports Medicine*.

[38] China Association of Chinese Medicine (2013). Chondromalacia patellae. *Rheumatism and Arthritis*.

[39] Blazina M. E., Kerlan R. K., Jobe F. W. (1973). Jumper’s knee. *Orthopedic Clinics of North America*.

[40] Zhang Z.-J., Chun-Long L., Ya-nan F. (2013). The research on the application of musculoskeletal ultrasonography to measure the thickness of patellar tendon. *Chinese Journal of Rehabilitation Medicine*.

[41] State Administration of Traditional Chinese Medicine (1994). *Criteria of Diagnosis and Therapeutic Effect of Diseases and Syndromes in Traditional Chinese Medicine*.

[42] Feng L. I., Zhu W.-L., Zhang H. (2015). Clinical study on 60 chronic knee-joint collateral ligaments inflammation patients treated by needle knife therapy combined with nerve block. *Shandong Medical Journal*.

[43] Zhang H.-B., Liu M., Wang L., Jiang T., Ren-you Z. (2012). Comparative analysis on domestic and foreign research of magnetic resonance imaging in the diagnosis of rheumatoid arthritis. *Chinese Journal of Tissue Engineering Research*.

[44] Dou R., Feng R. (2017). Diagnostic effect of musculoskeletal ultrasound in children with rheumatic osteoarthritis. *China Continuing Medical Education*.

[45] Jiang G. Y., Yao L. X. (2004). Chondromalacia patellae: further comprehension and clinical application. *Chinese Journal of Clinical Rehabilitation*.

[46] Zhang J., Ding J. (2015). The diagnosis and treatment of knee joint tuberculosis. *Chinese Journal of Bone and Joint Injury*.

[47] Wang C., Tianan J. (2015). Value of high-frequency ultrasound in the diagnosis of the knee joint tuberculosis. *Chinese Journal of Ultrasound in Medicine*.

[48] Wang T.-T., Sun W. P., Liang S. (2017). Meta-analysis of diagnostic value of procalcitonin in bone and joint infection of adult. *Chinese General Practice*.

[49] Coleman S., McQuade J., Rose J., Inderjeeth C., Carroll G., Briffa N. K. (2010). Self-management for osteoarthritis of the knee: does mode of delivery influence outcome?. *BMC Musculoskeletal Disorders*.

[50] Coleman S., Briffa N. K., Carroll G., Inderjeeth C., Cook N., McQuade J. (2012). A randomised controlled trial of a self-management education program for osteoarthritis of the knee delivered by health care professionals. *Arthritis Research & Therapy*.

[51] Ravaud P., Flipo R. M., Boutron I (2009). ARTIST (osteoarthritis intervention standardized) study of standardised consultation versus usual care for patients with osteoarthritis of the knee in primary care in France: pragmatic randomised controlled trial. *BMJ*.

[52] Ettinger W. H., Burns R., Messier S. P (1997). A randomized trial comparing aerobic exercise and resistance exercise with a health education program in older adults with knee osteoarthritis. The Fitness Arthritis and Seniors Trial (FAST). *JAMA*.

[53] Kovar P. A., Allegrante J. P., MacKenzie C. R., Peterson M. G., Gutin B., Charlson M. E. (1992). Supervised fitness walking in patients with osteoarthritis of the knee. A randomized, controlled trial. *Annals of Internal Medicine*.

[54] Fisher N. M., Gresham G. E., Abrams M. (1993). Quantitative effects of phy1sical therapy on muscular and functional performance in subjects with osteoarthritis of the knees. *Archives of Physical Medicine and Rehabilitation*.

[55] Jan M. H., Lai J. S. (1991). The effects of physiotherapy on osteoarthritic knees of females. *Journal of the Formosan Medical Association*.

[56] Chen J.-Z. (2001). *Current Physical Therapy*.

[57] Iversen M. D. (2012). Rehabilitation interventions for pain and disability in osteoarthritis: a review of interventions including exercise, manual techniques, and assistive devices. *Orthopaedic Nursing*.

[58] Roos E. M., Juhl C. B. (2012). Osteoarthritis 2012 year in review: rehabilitation and outcomes. *Osteoarthritis and Cartilage*.

[59] Yuan P.-W., Yang W., Wu-Lin K. (2016). The advances in rehabilitation research of osteoarthritis. *Rheumatism and Arthritis*.

[60] Xiao-Jie Y. U., Yi W. (2005). Application of exercise therapy in the treatment of knee osteoarthritis. *Chinese Journal of Physical Medicine and Rehabilitation*.

[61] Zhang S.-M. (2014). Application of exercise therapy in orthopedics rehabilitation. *The Journal of Traditional Chinese Orthopedics and Traumatology*.

[62] Huang T., Chang-Lin H. (1999). The effect of different movements on recovering the function of OA joints. *Chinese Journal of Rehabilitation*.

[63] Silva L. E., Valim V., Pessanha A. P. (2008). Hydrotherapy versus conventional land-based exercise for the management of patients with osteoarthritis of the knee: a randomized clinical trial. *Physical Therapy*.

[64] Lu M., Su Y., Zhang Y. (2015). Effectiveness of aquatic exercise for treatment of knee osteoarthritis: systematic review and meta-analysis. *Zeitschrift fur Rheumatologie*.

[65] Arthritis Australia (2015). *Medicines and Arthritis*.

[66] Stitik T. P., Altschuler E., Foye P. M. (2006). Pharmacotherapy of osteoarthritis. *American Journal of Physical Medicine & Rehabilitation*.

[67] National Prescribing Service (NPS) (2006). *Analgesic Options for Pain Relief*.

[68] Kidd B. L., Langford R. M., Wodehouse T. (2007). Arthritis and pain. Current approaches in the treatment of arthritic pain. *Arthritis Research & Therapy*.

[69] National Prescribing Service (NPS) (2006). *Analgesic Choices in Persistent Pain. Prescribing Practice Review*.

[70] Antman E. M., Bennett J. S., Daugherty A. (2007). Use of nonsteroidal antiinflammatory drugs. *Circulation*.

[71] Morelli V., Naquin C., Weaver V. (2003). Alternative therapies for traditional disease states: osteoarthritis. *American Family Physician*.

[72] American Academy of Orthopaedic Surgeons (2001). *AAOS Research Committee Fact Sheet. Osteoarthritis: Glucosamine and Chondroitin Sulfate*.

[73] Arthritis Australia (2004). *Fact Sheet: Glucosamine and Chondroitin*.

[74] He W., Kuang M., Zhao J. (2017). Efficacy and safety of intraarticular hyaluronic acid and corticosteroid for knee osteoarthritis: a meta-analysis. *International Journal of Surgery*.

[75] Bellamy N., Campbell J., Robinson V. (2005). *Intraarticular Corticosteroid for Treatment of Osteoarthritis of the Knee*.

[76] Chinese Rheumatology Association (2010). The guideline of diagnosis and treatment of osteoarthritis. *Chinese Journal of Rheumatology*.

[77] State Administration of Traditional Chinese Medicine (2017). *Criteria of Diagnosis and Therapeutic Effect of Diseases and Syndromes in Traditional Chinese Medicine*.

[78] Shi-Wei L. (2014). Reasonable choice in the traditional Chinese medicine for the treatment of osteoarthritis. *Heilongjiang Medicine Journal*.

[79] Li-Jun Z., Wen-Shan G., Xian-Guo L. (2016). Qizheng pain-relieving plaster inhibits the inflammatory pain and immune abnormality induced by complete freund’s adjuvant. *Chinese Journal of Pain Medicine*.

[80] Zheng Y.-X., Zhan H.-S., Zhang H. (2006). Qizheng pain-relieving plaster therapy for knee osteoarthritis -a randomized controlled clinical trial. *China Journal of Orthopaedics and Traumatology*.

[81] Shou-Hai H., Fei W., Xuan L. (2011). Study on the mechanisms of cupping therapy. *Chinese Acupuncture and Moxibustion*.

[82] Wang B., Xi-Ru L., Zhi-Hai H. (2011). Yang’s pricking-cupping therapy for knee osteoarthritis: a multi-center randomized controlled trial. *Chinese Acupuncture & Moxibustion*.

[83] China Association for Acupuncture and Moxibustion (2015). *Evidence-Based Guidelines of Clinical Practice with Acupuncture and Moxibusion: Knee Osteoarthritis*.

[84] Han-Zhang Z., Bai-Zhi L. (1999). *Acupotomy in Clinical Diagnosis and Therapy*.

[85] Nian-Zu L. U. (2012). *Lu’s Traumatology on Silver Needle Therapy*.

[86] Fu-Gen W. (2008). *Thermal Treatment of Soft Tissue Pain with Silver Needle*.

[87] Zhang Y.-L., Shao-yan Y. (2013). Massage technique in treatment of 97 cases of knee osteoarthritis. *Chinese Manipulation & Rehabilitation Medicine*.

[88] Koester M. C., Dunn W. R., Kuhn J. E., Spindler K. P. (2007). The efficacy of subacromial corticosteroid injection in the treatment of rotator cuff disease: a systematic review. *Journal of the American Academy of Orthopaedic Surgeons*.

[89] Coombes B. K., Bisset L., Brooks P., Khan A., Vicenzino B. (2013). Effect of corticosteroid injection, physiotherapy, or both on clinical outcomes in patients with unilateral lateral epicondylalgia: a randomized controlled trial. *JAMA*.

[90] Dakin S. G., Dudhia J., Smith R. K. (2014). Resolving an inflammatory concept: the importance of inflammation and resolution in tendinopathy. *Veterinary Immunology and Immunopathology*.

[91] Mohamadi A., Chan J. J., Claessen F. M. (2017). Corticosteroid injections give small and transient pain relief in rotator cuff tendinosis: a meta-analysis. *Clinical Orthopaedics and Related Research*.

[92] Charnoff J., Naqvi U. (2017). *Tendinosis (Tendinitis)*.

[93] National Clinical Guideline Centre (UK) (2014). *Osteoarthritis: Care and Management in Adults*.

[94] The Royal Australian College of General Practitioners (2009). *Guideline for the Non-Surgical Management of Hip and Knee Osteoarthritis*.

[95] McAlindon T. E., Bannuru R. R., Sullivan M. C. (2014). OARSI guidelines for the non-surgical management of knee osteoarthritis. *Osteoarthritis Cartilage*.

[96] Wang J. (2017). Efficacy and safety of adalimumab by intra-articular injection for moderate to severe knee osteoarthritis: an open-label randomized controlled trial. *Journal of International Medical Research*.

[97] Wehling P., Evans C., Wehling J. (2017). Effectiveness of intra-articular therapies in osteoarthritis: a literature review. *Therapeutic Advances in Musculoskeletal Disease*.

[98] Lisi C., Perotti C., Scudeller L. (2017). Treatment of knee osteoarthritis: platelet-derived growth factors vs. hyaluronic acid. A randomized controlled trial. *Clinical Rehabilitation*.

[99] Dai W. L., Zhou A. G., Zhang H. (2017). Efficacy of platelet-rich plasma in the treatment of knee osteoarthritis: a meta-analysis of randomized controlled trials. *Arthroscopy*.

[100] Bennell K. L., Hunter D. J., Paterson K. L. (2017). Platelet-Rich plasma for the management of hip and knee osteoarthritis. *Current Rheumatology Reports*.

[101] Barreto A., Braun T. R. (2016). A new treatment for knee osteoarthritis: clinical evidence for the efficacy of Arthrokinex autologous conditioned serum. *Journal of Orthopaedics*.

[102] Evans C. H., Chevalier X., Wehling P. (2016). Autologous conditioned serum. *Physical Medicine and Rehabilitation Clinics of North America*.

[103] Hochberg M. C., Altman R. D., April K. T. (2012). American College of Rheumatology 2012 recommendations for the use of nonpharmacologic and pharmacologic therapies in osteoarthritis of the hand, hip, and knee. *Arthritis Care & Research*.

[104] Nguyen C., Rannou F. (2017). The safety of intra-articular injections for the treatment of knee osteoarthritis: a critical narrative review. *Expert Opin Drug Saf*.

[105] Hadzic A., Houle T. T., Capdevila X. (2010). Femoral nerve block for analgesia in patients having knee arthroplasty. *Anesthesiology*.

[106] Zhang P., Li J., Song Y. (2017). The efficiency and safety of fascia iliaca block for pain control after total joint arthroplasty: a meta-analysis. *Medicine*.

[107] Kwofie M. K., Shastri U. D., Gadsden J. C. (2013). The effects of ultrasound-guided adductor canal block versus femoral nerve block on quadriceps strength and fall risk: a blinded, randomized trial of volunteers. *Regional Anesthesia and Pain Medicine*.

[108] Bali C., Ozmete O., Eker H. E. (2016). Postoperative analgesic efficacy of fascia iliaca block versus periarticular injection for total knee arthroplasty. *Journal of Clinical Anesthesia*.

[109] Wang X., Sun Y., Wang L. (2017). Femoral nerve block versus fascia iliaca block for pain control in total knee and hip arthroplasty: a meta-analysis from randomized controlled trials. *Medicine (Baltimore)*.

[110] San S., Aydin O. N., Turan Y. (2016). Which one is more effective for the clinical treatment of chronic pain in knee osteoarthritis: radiofrequency neurotomy of the genicular nerves or intra-articular injection?. *International Journal of Rheumatic Diseases*.

[111] Kesikburun S., Yaşar E. (2016). Uran a ultrasound-guided genicular nerve pulsed radiofrequency treatment for painful knee osteoarthritis: a preliminary report. *Pain Physician*.

[112] Qudsi-Sinclair S., Borras-Rubio E., Abellan-Guillen (2017). A comparison of genicular nerve treatment using either radiofrequency or analgesic block with corticosteroid for pain after a total knee arthroplasty: a double-blind, randomized clinical study. *Pain Practice*.

[113] Kirdemir P., Catav S., Alkaya Solmaz F. (2017). The genicular nerve: radiofrequency lesion application for chronic knee pain. *Turkish Journal of Medical Sciences*.

[114] Duymus T. M., Mutlu S., Dernek B., Komur B., Aydogmus S., Kesiktas F. N. (2017). Choice of intra-articular injection in treatment of knee osteoarthritis: platelet-rich plasma, hyaluronic acid or ozone options. *Knee Surgery, Sports Traumatology, Arthroscopy*.

[115] Calunga J. L., Menéndez S., León R. (2012). Application of ozone therapy in patients with knee osteoarthritis. *Ozone: Science & Engineering*.

[116] Yang S.-P., Guo-Sheng X. U., Huang J.-X. (2014). Clinical observation study of ozone perfusion combined with sodium hyaluronate injection in treatment of degenerative osteoarthritis. *Chinese Journal of Joint Surgery (Electronic Edition)*.

[117] Hashemi M., Jalili P., Mennati S. (2015). The effects of prolotherapy with hypertonic dextrose versus prolozone (intraarticular ozone) in patients with knee osteoarthritis. *Anesthesiology and Pain Medicine*.

[118] Radnovich R., Scott D., Patel A. T. (2017). Cryoneurolysis to treat the pain and symptoms of knee osteoarthritis: a multicenter, randomized, double-blind, sham-controlled trial. *Osteoarthritis and Cartilage*.

[119] Bennell K. L., Hunter D. J., Hinman R. S. (2012). Management of osteoarthritis of the knee. *BMJ*.

[120] De Windt T. S., Vonk L. A., Brittberg M. (2013). Treatment and prevention of (early) osteoarthritis using articular cartilage repair-fact orfiction a systematic review. *Cartilage*.

[121] Smith T. O., Sexton D., Mitchell P. (2011). Opening-or closing-wedged high tibial osteotomy: a meta-analysis of clinical and radiological outcomes. *Knee*.

[122] Nwachukwu B. U., Mccormick F. M., Schairer W. W. (2014). Unicompartmental knee arthroplasty versus high tibial osteotomy: United States practice patterns for the surgical treatment of unicompartmental arthritis. *Journal of Arthroplasty*.

[123] Carr A. J., Robertsson O., Graves S. (2012). Knee replacement. *The Lancet*.

[124] Liddle A. D., Pegg E. C., Pandit H. (2013). Knee replacement forosteoarthritis. *Maturitas*.

[125] Iacono F., Rspugli G. F., Brunl D. (2013). Arthrodesis after infected revision TKA: retrospective comparison of intramedullary nailing and external fixation. *HSS Journal*.

